# Evolution and Phylogenetic Analysis of Full-Length VP3 Genes of Eastern Mediterranean Bluetongue Virus Isolates

**DOI:** 10.1371/journal.pone.0006437

**Published:** 2009-07-30

**Authors:** Kyriaki Nomikou, Chrysostomos Ι. Dovas, Sushila Maan, Simon J. Anthony, Alan R. Samuel, Maria Papanastassopoulou, Narender S. Maan, Olga Mangana, Peter P. C. Mertens

**Affiliations:** 1 Arbovirus Molecular Research Group, Department of vector borne diseases, Institute for Animal Health, Pirbright Laboratory, Woking, Surrey, United Kingdom; 2 Department of Microbiology and Infectious Diseases, Faculty of Veterinary Medicine, Aristotle University of Thessaloniki, Thessaloniki, Greece; 3 Wildlife Disease Laboratory, San Diego Zoo Conservation Research, Escondido, California, United States of America; 4 Virus Laboratory, Institute of Infectious and Parasitic Diseases, Ministry of Rural Development and Food, Athens, Greece; University of Georgia, United States of America

## Abstract

*Bluetongue virus* (BTV) is the ‘type’ species of the genus *Orbivirus* within the family *Reoviridae*. The BTV genome is composed of ten linear segments of double-stranded RNA (dsRNA), each of which codes for one of ten distinct viral proteins. Previous phylogenetic comparisons have evaluated variations in genome segment 3 (Seg-3) nucleotide sequence as way to identify the geographical origin (different topotypes) of BTV isolates. The full-length nucleotide sequence of genome Seg-3 was determined for thirty BTV isolates recovered in the eastern Mediterranean region, the Balkans and other geographic areas (Spain, India, Malaysia and Africa). These data were compared, based on molecular variability, positive-selection-analysis and maximum-likelihood phylogenetic reconstructions (using appropriate substitution models) to 24 previously published sequences, revealing their evolutionary relationships. These analyses indicate that negative selection is a major force in the evolution of BTV, restricting nucleotide variability, reducing the evolutionary rate of Seg-3 and potentially of other regions of the BTV genome. Phylogenetic analysis of the BTV-4 strains isolated over a relatively long time interval (1979–2000), in a single geographic area (Greece), showed a low level of nucleotide diversity, indicating that the virus can circulate almost unchanged for many years. These analyses also show that the recent incursions into south-eastern Europe were caused by BTV strains belonging to two different major-lineages: representing an ‘eastern’ (BTV-9, -16 and -1) and a ‘western’ (BTV-4) group/topotype. Epidemiological and phylogenetic analyses indicate that these viruses originated from a geographic area to the east and southeast of Greece (including Cyprus and the Middle East), which appears to represent an important ecological niche for the virus that is likely to represent a continuing source of future BTV incursions into Europe.

## Introduction

*Bluetongue virus* (BTV) is the ‘type’ species of the genus *Orbivirus* within the family *Reoviridae* [1]. The BTV genome is composed of ten linear segments of double-stranded RNA (dsRNA), each of which codes for one of ten distinct viral proteins [2], [3]. The genome segments are packaged within an icosahedral capsid, ∼80 nm in diameter, composed of three concentric protein layers. The innermost ‘subcore’ shell is constructed from 12 decamers of VP3, surrounding the virus genome and viral transcriptase complexes composed of three minor structural proteins (VP1[Pol], VP4[CaP] and VP6[Hel]) that are attached to the inner surface of the sub-core layer [4]. The self-assembly of VP3 to form the BTV subcore, is thought to play a vital role in the early stages of virion assembly and replication [5], [6]. Once assembled, the subcore-shell provides a ‘scaffold’ for addition of 780 copies of VP7 (organized as 260 trimers) to form the core-surface layer, followed by addition of 60 trimers of VP2 and 120 trimers of VP5 to form the outer-capsid [3], [7], [8]. In BTV-infected cells there are also three distinct non-structural proteins (NS1, NS2 and NS3/NS3A) [2], [3].

VP3 interacts with both the minor proteins of the transcriptase complexes and the dsRNA of the viral genome, and consequently also helps to determine the internal structure of the particle [9]. This internal organization is considered to be important for the simultaneous synthesis of mRNA copies from the ten genome segments by the virus-core during replication within the infected cell [10]. The amino acid sequence of VP3, which determines the protein structure, therefore also determines both the overall size of the outer-layers and the internal organization of the BTV virus particle.

VP3 is encoded by genome segment 3 (Seg-3) and is composed of 901 amino acids, which form an elongated triangular protein that can be divided into apical, carapace, and dimerization domains [4], [11]. The amino acid sequence of VP3 (and the nucleotide sequence of Seg-3) is highly conserved within the species *BTV* and shows significant levels of conservation even between different *Orbivirus* species [1], [12], [13]. Indeed Seg-3 is one of the most conserved regions of the BTV genome [13], reflecting major structure/function constraints on VP3, as well as its internal position within the virus particle which protects it from exposure to neutralizing antibodies and ‘antibody selective pressure’. Despite these constraints, both Seg-3 and VP3 do show significant variations that reflect the geographic origins of the virus, dividing individual isolates into eastern and western groups/topotypes, and a number of further sub-groups [13], [14].

Bluetongue (BT) is an arthropod-transmitted disease of wild and domestic ruminants, which can spread rapidly under suitable circumstances. It has therefore been included in the ‘list of notifiable diseases’ (former list A) by the Office International des Epizooties (OIE) [15], and represents a major barrier to international trade in ruminants and their products, causing worldwide losses that were estimated in 1996 at US$3 billion per year [16].

Twenty four distinct serotypes of BTV have been detected around the world, between latitudes 40°S and 53°N (in the Americas, Africa, Asia and Australia and Europe), although not all of these serotypes occur in each region (www.reoviridae.org/dsRNA_virus_proteins/btv-serotype-distribution.htm). In 2008 a virus from goats in Switzerland (Toggenburg orbivirus) was provisionally identified as a further 25^th^ distinct serotype (BTV-25) [17]. Prior to 1998, outbreaks of BT had occasionally been detected in Europe, although in most cases these were relatively short lived (∼4–5 years) and involved a single BTV strain/serotype on each occasion (reviewed by Mellor [18]). However in 1998, a major series of BT incursions began in Europe, involving BTV-1, -2, -4, -6, -8 -9, -11 and -16 (see www.reoviridae.org/dsRNA_virus_proteins/outbreaks.htm; [13], [19], [20], [21]. These outbreaks started in the eastern Mediterranean region (in Greece) before spreading westwards across the Balkans and southern Europe (www.reoviridae.org/dsRNA_virus_proteins/ReoID/BTV-mol-epidem.htm; [18], [22,]. Although BTV incursions from the east and from North Africa were initially restricted to Mediterranean Europe, during August 2006 an outbreak caused by BTV-8 was detected in the Netherlands. This was the first time that BT had ever been recorded in northern Europe and represented the start of the largest single outbreak of BT on record, subsequently spreading across the whole of northern and central Europe 2008 [13], [18], [23].

The first recorded incursion of BT in Greece during 1979 was caused by BTV-4, on the island of Lesbos in the eastern Aegean Sea [24]. Although measures to control the disease were successful and Greece was subsequently declared BTV free in 1991, a new incursion began in the eastern Aegean region during 1998, followed by a number of major epizootics between 1998 and 2000 that involved a western topotype of BTV-4 and to a lesser extent, eastern topotypes of BTV-9 and BTV-16. In 2001 a further incursion was caused by an eastern topotype of BTV-1 [25], and in December 2008 both BTV-8 (western topotype) and BTV-16 (eastern topotype) were detected in Greece (see: www.reoviridae.org/dsRNA_virus_proteins/ReoID/virus-nos-by-country.htm#Greece).

Reassortment, genetic drift and founder effects during sequential passage of BTV between its insect and ruminant hosts, contribute to the diversification and independent evolution of each BTV segment [26]. Phylogenetic analyses based on the complete or partial nucleotide sequences of different segments have been carried out to determine the genetic relationships and/or geographic origins of different BTV isolates. These studies have involved genes encoding conserved proteins e.g. Seg-9, encoding VP6 [27], or the more variable Seg-10 encoding NS3/NS3A [28], [29], [30], [31] and the most variable outer-capsid proteins VP2 and VP5, encoded by Seg-2 and Seg-6 respectively [13], [32]–[38].

Comparisons of the full genome of the northern European strain of BTV-8 to other European isolates, have confirmed Seg-2 and -6 as the most variable components of the BTV genome [13], showing variations that correlate with both virus serotype and the geographic origins (topotype) of the virus isolate. Genome segments 7 and 10, encoding VP7 and NS3, show intermediate levels of variation, dividing isolates into a number of clades that show only partial correlation with virus topotype [13]. The remaining variations in Seg-7 and Seg-10 have as yet unconfirmed significance, although it has been suggested that they may relate to transmission of the virus by different insect vector species/populations [13], [39], [40,]. The remaining six genome segments (Seg-1, -3, -4, -5, -8 and 9; encoding VP1, VP3, VP4, NS1, NS2 and VP6, respectively) are all highly conserved, showing variations that correlate primarily with virus origin, dividing isolates into eastern and western topotypes and providing evidence of geographic subgroups, but no significant correlation with virus serotype (supplementary data, [13]).

Previous phylogenetic comparisons have evaluated variations in Seg-3 nucleotide sequence as way to identify the geographical origin (different topotypes) of BTV isolates [13], [14], [41]. These analyses have previously identified two major topotypes (eastern and western), represented by the Australasian, or American and African viruses respectively. However, an Australian isolate of BTV-15, and the Swiss isolate of BTV-25 appear likely to represent two further divergent groups or distinct topotypes. These genetic relationships have been inferred mostly using a partial (540 bp) fragment [14], [42], or in a few cases using a small number (one or two) of full-length Seg-3 sequences [41].

The study presented here analysed the full-length sequence of Seg-3 from twenty BTV isolates recovered during the 1979 and 1998–2001 epizootics of BT in eastern Mediterranean countries and the Balkans. Ten other reference strains, or field isolates of BTV from other locations, were included in these analyses. The sequence data generated were also compared to twenty four Seg-3 sequences from other BTV isolates that have previously been published, to elucidate the geographic origins of the European viruses and provide data concerning the relationships and evolution of Seg-3./p>

## Materials and Methods

### Virus isolates

**Figure 1:**
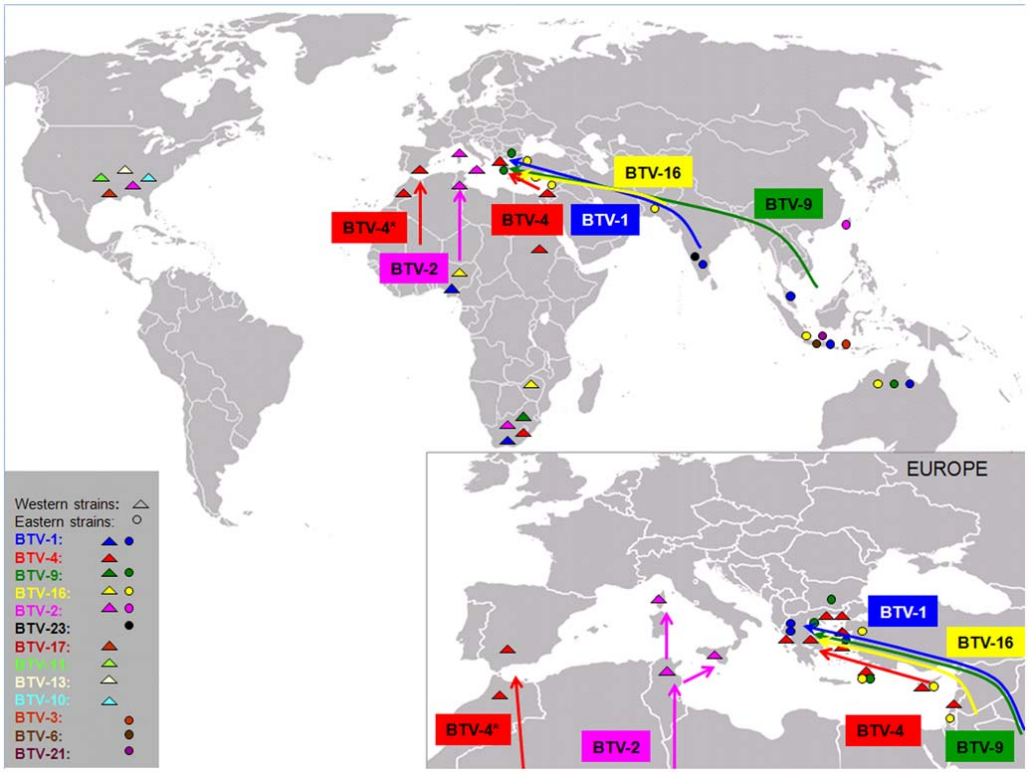
Map of geographical location of the isolates analysed in this study and the sources of BTV incursions in Europe.

Genome segment 3 was sequenced from thirty BTV isolates, including twenty isolates from Greece, Cyprus, Bulgaria, Turkey and Israel, six isolates from Spain, Morocco, Nigeria, Sudan, Malaysia and India and four reference strains (Table 1, Figure 1). The Greek BTV strains were isolated from ruminants during epizootics in 1979 and 1998–2001, using BHK-21 cells (ECACC 85011433) or embryonated chicken eggs, followed by passage in BHK-21 cells [43], [44]. Initial EDTA blood samples were taken from sick or sentinel animals, while spleen samples were taken at post-mortem. Isolates were serotyped by serum neutralization tests (SNTs) as described in the OIE Manual of Diagnostic Tests and Vaccines for Terrestrial Animals [45], using type specific antisera provided by the OIE Reference Laboratory at the Onderstepoort Veterinary Institute, Onderstepoort, South Africa. Each of these virus isolates is included in the reference collection at IAH Pirbright (see www.reoviridae.org/dsRNA_virus_proteins/ReoID/BTV-isolates.htm) and is identified here by their reference collection number.

**Table 1:** BTV field isolates sequenced in this study. Further details of the isolates can be obtained from the dsRNA virus reference collection (www.reoviridae.org/dsRNA_virus_proteins/ReoID/BTV-isolates.htm).

Abbreviations^1^	IAH^2^ dsRNA virus reference collection number	Passage History (E^3^/BHK-21^4^)	Seg-3 Sequence Accession Number	Area of isolation	Tissue	Host species
4/GR1/79	GRE 1979/01	E2/BHK6	DQ 186823	Lesvos, Greece	Spleen	Sheep
4/GR180/99	GRE 1999/01	E2/BHK4	DQ 186800	Rhodes, Greece	Blood	Sheep
4/GR631/99	GRE 1999/04	E2/BHK7	DQ 186801	Magnesia, Greece	Blood	Sheep
4/GR228/99	GRE 1999/07	E2/BHK4	DQ 186802	Evros, Greece	Blood	Goat
4/GR378/99	GRE 1999/09	E2/BHK7	DQ 186803	Chios, Greece	Blood	Bovine
4/GR395/99	GRE 1999/10	E2/BHK5	DQ 186804	Lesvos, Greece	Spleen	Sheep
4/GR279/99	GRE 1999/15	E2/BHK6	DQ 186809	Rodopi, Greece	Spleen	Sheep
4/GR1217/00	GRE 2000/07	E2/BHK3	DQ 186806	Arta, Greece	Blood	Sheep
9/GR408/99	GRE 1999/12	E2/BHK4	DQ 186805	Chalkidiki, Greece	Blood	Sheep
9/GR199/98	GRE 1998/01	E2/BHK9	DQ 186808	Rhodes, Greece	Blood	Sheep
16/GR308/99	GRE 1999/13	E2/BHK9	DQ 186821	Rhodes, Greece	Blood	Sheep
1/GR1245/01	GRE 2001/05	E1/BHK9	DQ 186822	Ioannina, Greece	Spleen	Sheep
1/GR15/01	GRE 2001/06	BHK8	DQ 186820	Grevena, Greece	Spleen	Sheep
1/GR1472/01	GRE 2001/07	BHK3	DQ 189807	Lesvos, Greece	Blood	Sheep
16/CYP/04	CYP 2004/01	Vero1/BHK4	DQ186798	Ammochostos, Cyprus	Blood	Sheep
4/CYP/04	CYP 2004/03	CPAE1/BHK6	DQ186799	Ammochostos, Cyprus	Spleen	Sheep
16/ISR/01	ISR 2001/18	E1/BHK4	DQ186814	Yokneam, Israel	Blood	Sheep
4/ISR/01	ISR 2001/01	E1/BHK2	DQ186813	Bat Slomo, Israel	Blood	Sheep
16/TUR/00	TUR 2000/01	E3/BHK9	DQ186827	Izmir, Turkey	-	-
9/BUL/99	BUL 1999/01	E1/BHK5	DQ186797	Bulgaria	-	-
4/SPA/03	SPA 2003/01	E1/BHK4	DQ186824	Menorca, Spain	Blood	Sheep
4/MOR/04	MOR2004/02	E1/BHK4	DQ186817	El Kebir, North Morocco	Blood	Sheep
1/NIG/82	NIG 1982/01	E1/BHK3	DQ 186818	Nigeria	-	-
4/SUD/83	SUD 1983/01	E1/BHK2	DQ186825	Sudan	-	-
1/MAY/87	MAY1987/01	E1/BHK2	DQ 186816	Kuala Lumpur, Malaysia	-	-
1/INDΙΑ/01	IND 2001/01	BHK6	DQ 186811	Chennai, India	-	*Culicoides* spp.
1/REF^5^	RSArrrr/01	E2/BHK9	DQ186792	OVI - South Africa	-	-
4/REF^5^	RSArrrr/04	BHK9	DQ186794	OVI - South Africa (Cyprus 1969)	-	-
16/REF^5^	RSArrrr/16	E2/BHK7	DQ186796	OVI - South Africa (Pakistan)	-	-
9/REF^5^	RSArrrr/09	E3/BHK8	DQ186795	OVI - South Africa	-	-

1 Serotype/origin (GR: Greece, NIG: Nigeria, MAY: Malaysia, INDIA: India, CYP: Cyprus, ISR: Israel, TUR: Turkey, BUL: Bulgaria, SUD: Sudan, SPA: Spain, MOR: Morocco, REF: Reference strain)/year of isolation.

2 Institute for Animal Health, Pirbright Laboratory, UK.

3 Number of passages in embryonated chicken eggs.

4 Number of passages in BHK-21 cell culture.

5 Reference strains of BTV were provided by the Onderstepoort Veterinary Research Institute, including strains of BTV-1 (1958) and BTV-9 (1942) from a South-African origin. However, the strains of BTV-4 and -16 were isolated in Cyprus (1969) and Pakistan (1960) respectively [83], [84].

### Full-length amplification of cDNA

dsRNA was extracted from pellets of BTV infected BHK-21 cells using Trizol® LS reagent (Invitrogen) as described by Attoui et al. [46] and used for amplification of full-length cDNAs using a self-priming ‘anchor-primer’, as described by Maan et al. [47]. Briefly a 35 base single-stranded self-priming oligonucleotide “anchor-primer” (5′ p-GAC CTC TGA GGA TTC TAA AC /iSp9/ T CCA GTT TAG AAT CC-OH 3′) was ligated to the 3′ (uncapped) termini of unfractionated BTV genomic dsRNA [47]. The ‘ligated’ dsRNA was denaturated, and then used for the synthesis of cDNA copies, primed by the base paired ‘hairpin’ structure of the anchor-primer. The ligation reactions were carried out at 37°C for 40 min, in a total volume of 40 µl, containing: 1.0 µg of dsRNA, 0.5 µl (0.25 µg) of anchor-primer (Integrated DNA Technology, USA), 2 µl of T4 DNA and RNA ligase buffers (Promega), 1 µl of RNasin (2 U/µl, Promega), 20 µl of Polyethylene Glycol (PEG, Sigma) and 0.5 µl each of T4 RNA and DNA ligase enzymes (at 10 U/µl and 3 U/µl respectively, Promega).

### Fractionation and purification of RNA segments

After ligation to the “anchor-primer”, the dsRNA genome segments of BTV were separated by electrophoresis in 0.9% agarose gels using Tris-Acetate/EDTA electrophoresis buffer (1× TAE), pH 8.0. The segments were excised from the agarose gels and extracted using the QIAquick® Gel extraction kit (Qiagen) according to the manufacturer’s instructions. Seg-2 and -3 were difficult to separate and were recovered as mixed preparations. The ligated RNA was eluted in 30 µl of RNase-DNase free water.

### cDNA first strand synthesis

The recovered, ligated-RNA (2.5 µl) was denatured and reverse transcription (RT) was performed at 50°C for 50 min using the Superscript™ III First-Strand Synthesis System for RT-PCR (Invitrogen), followed by 85°C for 5 min. The 20 µl reaction volume contained: 4 µl of 25 mM MgCL_2_, 1 µl of 10 mM each dNTP mix, 1 µl (200 U/µl) of Superscript™ III, 1 µl (40 U/µl) of RNaseOUT™, 2 µl of 0.1 M DTT, 2 µl of 10X RT buffer, 3 µl of RNase-DNase free water and 6 µl of denaturated RNA. After RT, 1 µl of RNase H (2 U/µl, Invitrogen) was added to the reaction, which was incubated at 37°C for 25 min, followed by denaturation at 95°C for 5 min. The primary cDNA strands were re-annealed by lowering the temperature from 95°C to 80°C, at a rate of 1°C per min, then to 50°C at a rate of 1°C per 3 min and incubation at 50°C for 10 min. The completed cDNA copies were diluted by addition of 50 µl of RNase-DNase free water and stored at −20°C.

### PCR amplification of cDNAs

Amplification of cDNAs was performed using the primer 5-15-1 (5′-GAGGGATCCAGTTTAGAATCCTCAGAGGTC-3′) [47] and the Triplemaster™ PCR system (Eppendorf) in a 20 µl reaction mixture containing 2 µl of the annealed primary cDNA product, 2 µl of 10X HiFidelity Buffer, 0.4 µl of dNTPs mix (10 mM each), 0.25 µl (5 U/µl) of Triplemaster™ enzyme, 1 µl of 100 µM 5-15-1 primer, and 15.4 µl of RNase-DNase free water. After denaturation at 94°C for 2 min, amplification was carried out for 35 cycles of 95°C for 20 sec and 68°C for 4.5 min followed by a final extension step at 72°C for 10 min.

### Sequencing and sequence analysis

The amplified RT-PCR products were analysed by 0.9% agarose gel electrophoresis for 6 hours, in TAE buffer. This was carried out at +4°C, to improve the separation of Seg-2 and Seg-3 and recovered from the gel using the GFX™PCR DNA and Gel Band Purification Kit (Amersham Biosciences) according to the manufacturer’s instructions. The purified DNA was sequenced in both directions using BTV-specific terminal ‘phased’ primers PHX-F (5′-AGAATCCTCAGAGGTCGTTAAA-3′) and PHX-R (5′-AGAATCCTCAGAGGTCGTAAGT-3′) [37], [47], as well as walking-sequencing primers (data not shown). Sequencing was carried out using the “Quick Start Mix” kit (CEQ DTCS Beckman Coulter) and reactions were run on a Beckman CEQ™ 8000 Automated Capillary Sequencer according to the manufacturer’s instructions. The resulting sequence data were analysed using BioEdit Sequence Alignment Editor (version 7.0.5.2) [48]. The Seg-3 sequences obtained for this study were all deposited to GenBank and the accession numbers obtained are provided in Table 1.

The Seg-3 sequences generated (Table 1), were aligned with other published sequences (Table 2) using ClustalX version 1.8 [49]. They were further analyzed using DnaSP version 4.10.9 [50] for calculating nucleotide diversity ‘Pi’ (the average number of nucleotide differences per site between two sequences). ‘Dxy’ represents the mean distance between two sequence groups (the average number of nucleotide substitutions per site between the two populations) [51].

**Table 2:** BTV isolates and published Seg-3 sequences used for sequence analysis. Further details of the isolates can be obtained from the dsRNA virus reference collection (www.reoviridae.org/dsRNA_virus_proteins/ReoID/BTV-isolates.htm).

Abbreviations^1^	IAH^2^ dsRNA virus reference collection number	Seg-3 Sequence Accession Number	Area of isolation
16/ZIM/03	ZIM 2003/10	DQ186828	Zimbabwe
16/NIG/82	NIG 1982/10	DQ186819	Nigeria
2/TUN/00	TUN 2000/01	DQ186826	Tunisia
2/ITL/02	ITL 2002/07	DQ186815	Sicily - Italy
23/INDIA/98	IND 1998/01	DQ186810	Bangalore, southern India
16/INDO/91	ISA 1991/01	DQ186812	Indonesia
9/AUS/85	AUS 1985/01	DQ186790	Australia
16/AUS/86	AUS 1986/02	DQ186791	Australia
2/CORS/00	-	AY124371	Corsica-France
2/OnaAUSA/95	-	S78452	USA
17/USA/97a	-	AF017280	USA
11/USA/94	-	L19968	USA
17/USA/85	-	M32722	USA
13/USA/94	-	L19969	USA
17/USA/97	-	AF017281	USA
10/USA/85	-	NC006014	USA
1/AUSAsia	-	AF529048	AUSTRALASIA
3/INDO/90	-	AF529045	West Java-Indonesia
2/TAIW/03	-	AY493688	Kinmen island -Taiwan
6/INDO/90	-	AF529047	West Java-Indonesia
21/INDO/90	-	AF529046	West Java-Indonesia
1/AUS/79	-	AY322428	Australia
1/INDO/90	-	AF529044	West Java-Indonesia
2/REF	RSArrrr/02	DQ186793	OVI - South Africa

1 Serotype/origin (ZIM: Zimbabwe, NIG: Nigeria, TUN: Tunisia, ITL: Italy, INDIA: India, AUS: Australia, CORS: Corsica, USA: USA, AUSAsia: Australasia, INDO: Indonesia, TAIW: Taiwan, REF: Reference strain)/year of isolation.

2 Institute for Animal Health, Pirbright Laboratory, UK.

### Phylogenetic and positive selection analysis

**Figure 2:**
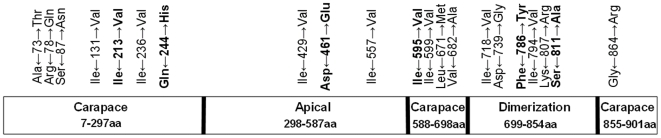
Schematic representation of VP3, showing the location of parsimony-informative sites found among viral isolates. The three domains of VP3 (apical, carapace, and dimerization) according to Grimes et al., (1998) are indicated. Positions 213, 244, 461, 595, 786, and 811 that are conserved within each lineage are indicated in bold and correspond to Ile, Gln, Asp, Ile, Phe, Ser for the ‘west’ lineage and Val, His, Glu, Val, Tyr, Ala for the ‘east’.

The best nucleotide substitution model selected by Modeltest 3.8 for the Seg-3 coding region sequence data set, was the General Time Reversible with gamma distributed rates across sites and a fraction of sites assumed to be invariable (GTR + I + Γ) [52]. Recombination can adversely affect the power and accuracy of phylogenetic reconstruction [53] and may result in higher rates of false positives in maximum likelihood tests for positive selection [54]. Therefore, the data was initially checked for evidence of recombination, using the Genetic Algorithm for Recombination Detection (GARD), www.datamonkey.org/GARD, [55]. The aligned sequence data set for the whole coding region of Seg-3 from 54 isolates (Table 1 and 2) was used for phylogenetic analyses. The best fitting nucleotide substitution model was selected by Modeltest 3.8 program [52] using the Akaike Information Criterion (AIC). Maximum likelihood (ML) phylogenetic trees were constructed with PhyML using a hill-climbing algorithm that adjusts tree topology and branch lengths simultaneously [56]. The reliability of the phylogenetic hypothesis was assessed using non-parametric bootstrap (NPB) analysis. The resulting tree was midpoint rooted using MEGA version 3.1 [57]. Phylogenetic analyses of the 5′ (nucleotides 36 to 908), middle (nucleotides 909 to 1778) and 3′ (nucleotides 1779 to 2648) regions of the Seg-3 were conducted in a similar manner. These regions have similar lengths and encode amino acid regions 7–297, 298–587 and 588–877 respectively that correspond to specific domains of VP3 (Figure 2).

**Figure 3:**
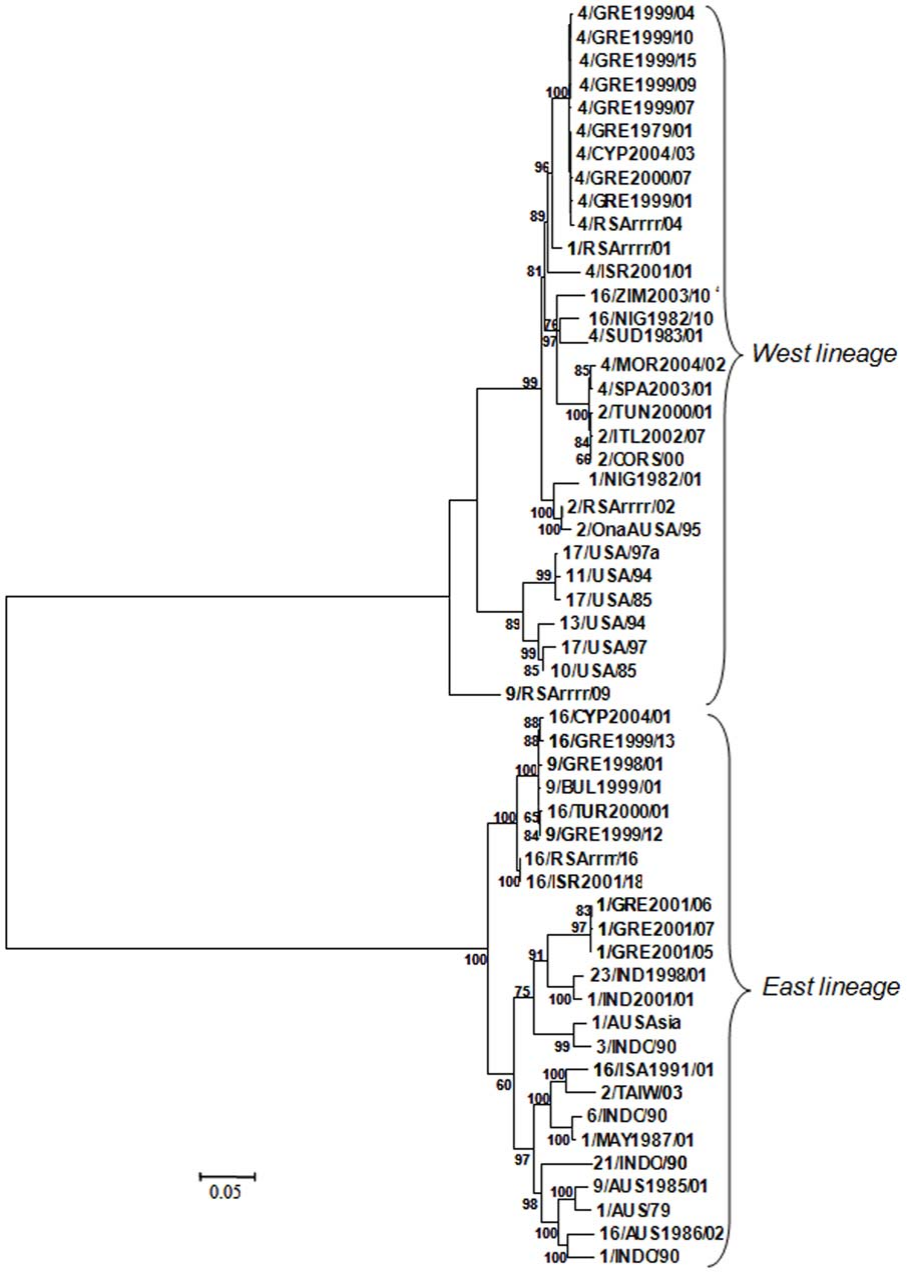
Phylogenetic tree constructed using maximum likelihood analyses using the whole coding region of BTV Seg-3 and the GTR + I + Γ model. The viruses included are listed intables 1 and 2. Isolate abbreviations: Serotype/origin (GRE: Greece, ISR: Israel, ZIM: Zimbabwe, NIG: Nigeria, SUD: Sudan, MOR: Morocco, SPA: Spain, TUN: Tunisia, ITL: Italy, CORS: Corsica, USA: USA, CYP: Cyprus, BUL: Bulgaria, TUR: Turkey, IND: India, AUSAsia: Australasia, INDO: Indonesia, TAIW: Taiwan, MAY: Malaysia, AUS: Australia, ISA: Indonesia, RSA: Reference strain)/year of isolation or IAH dsRNA virus reference collection number. The tree is midpoint rooted for purposes of clarity. The numbers indicated on branches are non-parametric bootstrap (NPB) probabilities and only values with P>0.6 are shown.

Positive selection analysis at single amino acid sites was performed separately for the eastern (Australasian) and western (N^th^ American/S^th^ African) lineages. For each of these aligned data sets we estimated the rates of non-synonymous and synonymous changes at each site, using likelihood-based methods as implemented in the on-line Datamonkey server (http://www.datamonkey.org; [58], [59] (Figure 3). These analyses used: i) a conservative single-likelihood ancestor-counting (SLAC) method, which is related to that of Suzuki–Gojobori [60] and ii) a fixed-effects likelihood (FEL) method that was used to directly estimate non-synonymous and synonymous substitution rates at each site (this method is more appropriate for data sets with a moderate number of sequences) [58]. For these analyses an optimal model of nucleic acid selection was selected by the methods available on the Datamonkey website using neighbour joining phylogenetic trees. The SLAC method was also used to calculate the global ratio of non-synonymous substitutions per non-synonymous site (d_N_) to synonymous substitutions per synonymous site (d_S_) (expressed as *d*_N_/*d*_S_) and estimate the 95% confidence interval for each dataset.

## Results

### Sequence and phylogenetic analysis of Seg-3

The BTV Seg-3 sequences analysed were all 2772 bp in length, containing a single open reading frame of 2706 bp, from nucleotide 18 to 2723 encoding VP3 protein of 901 amino acids (aa). In all sequences, an initiation codon (ATG) was located at positions nt 18–20, with a termination codon (TAG) at positions nt 2721–2723. In each case the coding region was flanked by 17 bp 5′ and 49 bp 3′ non-coding regions (NCRs) and the nucleotide sequence alignment showed no evidence of insertions or deletions. The majority of the nucleotide changes detected were silent, occurring in the third base position of each codon. The Seg-3 sequence was highly conserved, even between isolates belonging to different serotypes. No evidence for recombination was found using the GARD algorithm.

Comparisons of VP3 amino acid sequences from the fifty four BTV isolates revealed an extremely high overall level of conservation, with polymorphic positions representing only 7.77% of the amino acid sites. All of the VP3 sequences contained a low content of charged amino acids (less than 24.87%) and a high content of hydrophobic and non-polar residues. The hydrophobic residues constituted 46.18% of the protein. Two amino acids (residues 500 (M) and 502 (R)), which were previously identified as critical for assembly of virus cores [12], were also conserved in all of the sequences described here. Residues 307–328 of VP3 (T2)A, which lie immediately within the VP3 (T2) layer [9] and form strong interactions with the dimeric ‘capping’ enzyme VP4(CaP) [61], were also conserved in all isolates.

When the two main geographic lineages of VP3 were considered separately, the percentage of polymorphic positions was even lower (4.1% in western-topotype and 3.77% in the eastern-topotype). Twenty one parsimony-informative sites were identified (positions containing at least two types of amino acids and at least two of them occurring with a minimum frequency of two). Most of these substitutions were conservative with Ile↔Val being the most frequent. Positions 213, 244, 461, 595, 786, and 811 were different between, but conserved within the eastern and western topotypes (Figure 2).

ML phylogenetic analysis of Seg-3 nucleotide sequences also demonstrated the existence of two divergent lineages/topotypes, which correspond to the ‘eastern’ (Australasian) and ‘western’ (N^th^ American/S^th^ African) groups of BTV isolates (Figure 3). Nucleotide diversity within each lineage was similar (eastern Pi = 0.0779, SD = 0.003; western Pi = 0.064, SD = 0.0065). The overall percentage nucleotide similarity was 78.9%–99.8% within the eastern group and 89.3%–99.9% within the western group. The mean distance between these lineages, expressed as the average number of nucleotide substitutions per site between the two populations, was Dxy = 0.1919.

Seg-3 from each of the Greek isolates (GRE1979/01, GRE1999/01, GRE1999/04, GRE1999/07, GRE1999/09, GRE1999/10, GRE1999/15, GRE2000/07) and the Cypriot isolate (CYP2004/03) of BTV-4, belongs to the western group. These sequences are closely related to the reference strain RSArrrr/04, which was also isolated in Cyprus (1969) (Figure 3), showing high overall nucleotide similarity of 99.5%–99.9% (Pi = 0.0027, SD = 0.0005) with thirty two nt changes, but only one aa change between the more recent isolates and GRE1979/01 or RSArrrr/04 (1969). These BTV-4 isolates represent an eastern Mediterranean cluster that is closely related to the reference strain of BTV-1 (RSArrrr/01) and the Israeli isolate of BTV-4 (ISR2001/01). However, Seg-3 from this group of viruses is clearly distinct from that of the BTV-2 (TUN2000/01, ITL2002/07) and BTV-4 (SPA2003/01, MOR2004/02) isolates from the western Mediterranean region, even though these strains also belong to the western topotype.

In contrast, Seg-3 from the rest of the BTV-1, -9 and -16 isolates from Greece, Cyprus, Bulgaria, Turkey and Israel, all belong to the eastern topotype, but cluster into two separate lineages (sub-groups) both supported by high bootstrap values (100% and 60%). One lineage includes the BTV-16 isolates from Pakistan (RSArrrr/16) and Israel (ISR2001/18) as well as BTV-16 isolates (TUR2000/01, CYP2004/01, GRE1999/13) and BTV-9 isolates (GRE1999/12, GRE1998/01, BUL1999/01) from Turkey, Cyprus, Bulgaria, and Greece, showing overall nucleotide similarity of 97.7%–99.9% (Pi = 0.0054, SD = 0.0009). However, the three Greek BTV-1 isolates (GRE2001/05, GRE2001/06, GRE2001/07), along with the isolates from India (BTV-23/IND1998/01, BTV-1/IND2001/01), Indonesia (BTV-3/INDO/1990) and Astralasia (BTV-1/AUSAsia) form a distinct lineage (75% bootstrap support) that also shows overall nucleotide similarity 92.3%–94.4% (Pi = 0.0012, SD = 0.0004), with the Indian and Greek isolates being most closely related (overall nucleotide similarity 94%–94.4%).

A third distinct lineage was detected within the eastern Seg-3 topotype, containing isolates from south-east Asia and Australia (BTV-16/ISA1991/01, BTV-2/TAIW/2003, BTV-6/INDO/1990, BTV-1/MAY1987/01, BTV-21/INDO/1990, BTV-1/INDO/1990, BTV-9/AUS1985/01, BTV-16/AUS1986/02 and BTV-1/AUS/1979) supported by high bootstrap values (97%).

Phylogenetic analysis of the 5′ region of the Seg-3 which encodes part of the carapace domain (7-297 aa) gave almost identical topologies and bootstrap support with those inferred using the whole Seg-3 coding region. However, significant divergence in topology and bootstrap support was revealed in the trees inferred using the middle or the 3′ coding region of Seg-3 (Figure S1, S2 and S3 - Supporting information).

### Positive selection

Positive selection analysis was performed separately for the western and eastern lineages of BTV. The SLAC and FEL methods did not identify any sites in the western lineage which gave evidence of positive selection significant at the *p*<0.1 level. In the eastern lineage, codon site 78 (Gln→Arg) (p = 0.09) was identified by the FEL method as being influenced by positive selection.

The global estimate of *d*_N_/*d*_S_ was 0.015 (95% likelihood profile based confidence intervals, CI = 0.01–0.02) for the western lineage and 0.013 (95% CI = 0.01–0.018) for the eastern lineage, indicating strong purifying selection. A high number of negatively selected codons were identified in both lineages. SLAC identified 147 negatively selected sites in the western lineage and 203 in the eastern, while FEL identified 338 and 429 sites significant at the *p*<0.1 level, respectively.

### Near terminal non-coding regions (NCRs)

**Figure 4:**
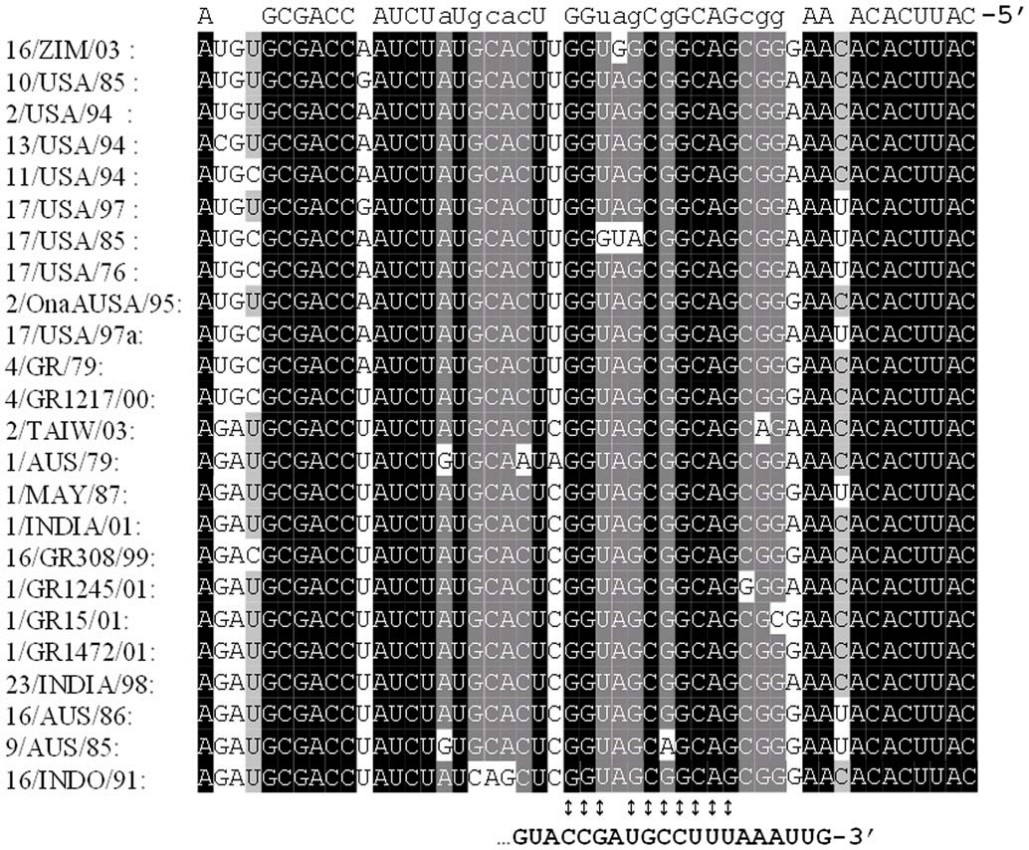
Sequence alignment of the 5′ terminal NCRs determined, indicating base pairing with the 3′ ends of the RNA transcripts. Only divergent sequences are shown.

Comparisons of the near terminal NCRs of Seg-3 from the BTV isolates (Table 1), along with other published sequences (Table 2), showed no differences in their length. The NCRs showed a higher level of conservation overall than the coding region, suggesting an important functional role. The 17 bp upstream-NCR was identical in all of the isolates compared, while the 49 bp downstream-NCR showed only low levels of diversity within each lineage (eastern Pi = 0.049, SD = 0.009; western Pi = 0.044, SD = 0.008). The mean distance between lineages was Dxy = 0.121. In each case the conserved terminal hexanucleotides were in complete agreement with the sequences reported for BTV (+ve 5′-GUUAAA------ACUUAC-3′) [1], [62]. Comparative sequence analysis of the terminal NCRs, also confirmed the existence of short inverted repeat sequences [3], indicating base pairing between the 5′ and 3′ ends of the RNA transcripts (Figure 4).

## Discussion

In order to make meaningful comparisons of sequence data derived from tissue culture adapted isolates of BTV, it is important to know the effect that virus isolation and multiple passage in embryonated chicken eggs and mammalian cell cultures may have on the RNA sequence of individual genome segments. Although only limited sequence data are available for the live vaccine strains of BTV, in some cases it has been possible to compare their sequences to those of the reference strains from which they were derived. Indeed, initial comparisons of the South African reference and vaccine strains of BTV-6 and BTV-9, suggest that multiple passages in embryonated chicken eggs (60 or 70 passages respectively) and in BHK cells (7 or 11 passages respectively) as well as multiple plaque selections [63], have led to few if any changes in RNA sequence (<0.4% variation in Seg-2, <0.18% variation in Seg-3 ) [64], [65].

In the study presented here, full length nucleotide sequences were generated for Seg-3 of twenty isolates of BTV, from the eastern Mediterranean region (from Cyprus, Bulgaria, Turkey Israel and Greece 1979, 1998–2001), as well as ten other reference strains and isolates, from other parts of the world (Figure 1). These data were compared to sequences for Seg-3 from 24 further BTV strains that have already been published [14], [42], [66]–[70], providing an analysis of the largest set of full-length BTV Seg-3 sequences to date.

Comparison of the terminal NCRs of Seg-3 showed a high level of sequence conservation (the 5′ NCR was 100% conserved, although some variability was evident in the 3′ NCR (7.7% mean overall variability), as reported in earlier studies [13], [14], [42]. These comparisons also showed short inverted repeat sequences that are consistent with base pairing between the 5′ and 3′ ends of the mRNA transcripts (Figure 4) [3]. The formation of these partial duplex structures between the terminal NCRs may influence the secondary structure of the mRNA and consequently induce formation of specific stem-loop structures, which it has been suggested can interact with NS2 and may be involved in the selection and packaging of the mRNA segments during virus assembly [71], [72], [73]. Conserved terminal hexanucleotides and adjacent inverted repeats are present in the NCRs of all 10 BTV genome segments, suggesting that they provide an important role as recognition signals for initiation of transcription and/or RNA replication and packaging [62]. It is also possible that they play a role in the efficient translation of viral mRNAs.

Although Seg-2 is the most variable region of the BTV genome, a high level of conservation was observed between isolates of the same serotype circulating in small geographic areas, suggesting a lack of diversifying selection (diversifying selection drives rapid differentiation of gene sequences and is one of the main forces behind adaptive evolution) (for example BTV-11 in Northern Colorado and BTV-4 in Greece and Turkey) [31], [74].

As an indicator of the action of natural selection in gene sequences the ratio of nonsynonymous (amino acid-altering) to synonymous substitutions (silent) (*d*_N_/*d*_S_) is versatile and widely used and provides an estimate of the selective pressure at the protein level [75]. A value of *d*_N_/*d*_S_>1 means that non-synonymous mutations offer fitness advantages to the protein and have higher fixation probabilities than synonymous mutations (diversifying or positive selection). On the other hand, *d*_N_/*d*_S_ values close to 0 mean that the protein is essentially conserved at the amino acid level (purifying or negative selection imposed by functional constraints) and *d*_N_/*d*_S_ = 1 corresponds to neutral evolution. Global *d*_N_/*d*_S_ values provide a means of assessing adaptive evolutionary pressures acting on a gene [75]. Approximately 36 to 43% of VP3 sites in both eastern and western BTV lineages appeared to be evolving under purifying selection, with *d*_N_/*d*_S_ values of 0.013 and 0.015 respectively indicating ‘strong’ purifying selection. As expected, given its critical role in core formation and function [1], [6] VP3 is subjected to powerful constraints and therefore appears unable to tolerate high levels of sequence variation. The genetic distance between the eastern and western lineages of Seg-3 (long internal tree branches, Figure 3) was high compared to the intra-lineage distances (short external tree branches, Figure 3). This could be an indication that in VP3, multiple amino acid substitutions are accommodated in a coordinated manner that preserves protein function and structure. Moreover, the fact that aa positions 213, 244, 461, 595, 786, and 811 are conserved within each lineage (Figure 2) suggests a possible co-variation of these amino acid positions, although this requires further experimental examination.

Rapid divergence of the NS3 and VP2 genes of BTV during experimental transmission between sheep, cattle and *C. sonorensis*, have indicated that the virus is capable of rapid sequence changes under appropriate conditions [26]. This suggests that the relatively low rates of BTV evolution observed in nature are not simply due to the innate properties of the genome replication mechanism (eg. a high fidelity viral polymerase or proof-reading). However, different selection forces and evolutionary constraints appear likely to restrict the evolution of the different BTV genome segments, with reassortment, genetic drift and founder effects contributing to the gradual overall diversification of individual BTV strains [26].

Low levels of evolutionary change are previously been recorded in other RNA arboviruses [76]. The requirement for replication in diverse host species (e.g. mammals and invertebrates) imposes important functional constraints and strong purifying-selection, minimising genetic drift and reducing the rates of nucleotide substitution [26], [77]. However rapid phenotypic changes in the virus due to mutations and founder effects may occasionally be induced as the virus passes through genetic bottleneck during each sequential passage between ruminant hosts and insect vectors [26]. BTV evolves with these constraints, potentially explaining how it can circulate for many years in a single geographic area without significant change, resulting in little or no evidence of temporal variation during phylogenetic comparisons.

BTV-VP3 exhibits fewer evolutionary changes than VP2, VP5, VP7 or NS3 [13]. This is reflected by the relatively small genetic distances that were detected between the Greek isolates of BTV-1, and the isolates of BTV-9 or BTV-16 (irrespective of their year of isolation: 1999–2004), or their different origins within the eastern Mediterranean region (Cyprus, Greece, Bulgaria or Turkey). A low nucleotide diversity, <0.4% (Pi = 0.0027, SD = 0.0005) was also observed in Seg-3 between the eight Greek and one Cypriot isolate of BTV-4. Maximum likelihood (ML) phylogenetic analysis showed small genetic distances and indicated a lack of a temporal distribution, despite the 25 year period covered by these BTV-4 isolates (Figure 3).

Overall negative selection appears to be the major evolutionary force affecting nucleotide diversity and evolution rates of Seg-3. A low level of nucleotide diversity has also been reported for Seg-10 sequences of the same Greek isolates [29], [78], indicating that conservation of these eastern Mediterranean BTV-4 isolates is not limited to VP3.

Geographic separation has resulted in divergence in BTV Seg-3 sequences that is consistent with the evolution of distinct viral populations [13], [14]. Phylogenetic analysis of Seg-3 therefore provides a potential mechanism to determine the geographic origins of the BTV incursions into South Eastern Europe. ML phylogenetic comparisons using the best fitted model of nucleotide substitution, separated the Seg-3 sequences of the Greek, Cypriot, Bulgarian, Turkish and Israeli isolates into three distinct clusters/lineages, one containing isolates of BTV-4 from a western-origin/western-topotype, and two other clusters/lineages containing isolates of BTV-1, -9 and -16 from an eastern origin/within the eastern-topotype.

Similar eastern and western origins for the Greek isolates were previously inferred by analysis of Seg-10 [29]. Based on Seg-3 analyses two different subclusters of closely related BTV-4 isolates circulating in the Mediterranean region were revealed. One includes closely related Greek isolates of BTV-4 along with a BTV-4 isolate from Cyprus 2004 and the reference strain RSArrrr/04 (which derives from the BTV-4, ASOT 1 strain isolated in 1969 in Cyprus). These isolates were also closely related to a BTV-4 isolate from an outbreak in Israel during 2001, indicating the existence of a BTV-4 sub-lineage in eastern Mediterranean region. The second subcluster included BTV-4 isolates from Morocco, Spain, Tunisia, Italy and Corsica that appear to have a western Mediterranean origin.

The results obtained with Seg-3 confirm the existence of at least two separate gene pools of BTV-4 in the Mediterranean basin. Potgieter et al (2005)[22] analyzed Seg-2 sequences of Greek BTV-4 isolates, along with those originating from western Mediterranean countries and found two different groups of isolates circulating in this region. This indicates two different sources for BTV-4 incursions in European countries, one from the southeast and the other from the southwest (North Africa) (Figure 1). Similar conclusions resulted after a more detailed sequence analysis based on five genome segments (Seg-2, -7, -8, -9 and -10) of BTV-4 isolates from different Mediterranean countries [31]. These analyses all support the conclusion that two subclusters of BTV-4 have been circulating in southern Europe, one causing incursions into Greece (1979, 1999–2000) and the second causing epizootics into Italy, Spain, Corsica and Morocco from 2003 onward. Phylogenetic analysis has indicated a common origin for the Portuguese BTV-4 isolates (2004–2006) and those from Sardinia and Corsica (2003), indicating that viruses belonging to this second cluster had recently spread to Portugal [30].

The Greek BTV-1, -9 and -16 isolates segregate into two branches within the eastern topotype, reflecting different geographic origins. The Greek BTV-1 isolates are more closely related to other eastern BTV-1 strains, particularly those from India. Circulation of BTV-1 had not previously been detected in the Mediterranean region [19]. Despite a lack of serological evidence for BTV-1 infection in Turkey during a survey in the early 1980s [79], the Greek strain of BTV-1 may have originated from eastern Turkey or the Middle East. However the BTV-8, BTV-6, and BTV-11 outbreaks in northern Europe, which started during 2006 and 2008, were not derived from outbreaks in contiguous territories. These events indicate that exotic strains of the virus can originate from distant sources, suggesting that the eastern Mediterranean strain of BTV-1 could have been introduced directly from India (Figure 1). This serotype persisted only for a relatively short period (2001) in Greece and did not spread to other European countries.

Interestingly, the BTV-9 and -16 isolates analysed form a separate sub-lineage that included isolates from Greece (BTV-16 and -9), Cyprus (BTV-16), Bulgaria (BTV-9), and Turkey (BTV-16) along with more distantly related isolates from Israel (BTV-16) and the reference strain RSArrrr/16 originating from Pakistan [63]. BTV-9 and -16 isolates from Australia and Indonesia analysed group to a separate sublineage. European strains containing Seg-3 belonging to this sub-lineage appear to have arrived from the Middle East. Evidence for BTV-9 infection was provided by a serological survey conducted in western and southern Anatolian Turkey during 1979–1980 [79]. Outbreaks caused by this BTV type have been reported for several years in Anatolian Turkey, Syria, Jordan and Israel in the period 1978–1981 [19]. The first incursion of BTV-9 in Europe was reported in Greece (Rhodes) during 1998. This serotype was also detected during a second outbreak on mainland Greece, Bulgaria and Turkey during 1999 [19]. High levels of nucleotide identity in Seg-2 between the 1999 Greek and 2001 Italian BTV-9 isolates (>99%) indicate that this strain also spread to Italy [22].

The first European strain of BTV-16 was isolated in 1999, from outbreaks on the Greek islands in the eastern Aegean Sea [25]. A close phylogenetic relationship was detected between the Greek, Cypriot and Turkish isolates of BTV-16, with almost identical Seg-3 sequences (99.4% similarity) to those isolates from Israel and Pakistan (bootstrap values 100%), again suggesting that the virus arrived in Europe from the east, possibly via Turkey (Figure 1). Analyses of Seg-2 from the European BTV-16 isolates have indicated an eastern origin [22], [80] as well as a common origin with the South African BTV-16 vaccine strain (derived from the reference strain from Pakistan). Analyses of French (Corsica), Italian and Israeli field isolates of BTV-16 indicate a recent common ancestry with the vaccine strain and have demonstrated reassortment with the BTV-2 vaccine strains, involving genome segment 5 (NS1 gene) [22], [81].

Since 1995, a live attenuated BTV-16 vaccine strain has been used as part of a live pentavalent vaccine (prepared in South Africa) containing BTV-2, -4, -6, -10 and -16, which was given annually on a voluntary basis to a small number of susceptible sheep in Israel [82]. Since 2004, BTV-16 has been removed from this vaccine, for safety reasons. The high sequence identity (99.4%) of Seg-3 from isolates belonging to different serotypes (BTV-9 and -16), was also reported for Seg-10 of the same Greek isolates [29] and indicates that reassortment involving replacement of Seg-2, or Seg-3 and Seg-10 has occurred.

There is considerable evolutionary pressure on the orbiviruses to maintain a stable and enzymatically functional core-particle. The role of the VP3 protein therefore appears to be mainly structural, and it is less affected by the nature of the hosts' species than the outer capsid proteins. The evolution of VP3 appears to be driven primarily by random genetic drift coupled with strong negative selection, resulting in low evolution rates. As a consequence Seg-3 provides a valuable analytical target to trace the origins of BTV strains during epizootics. However, the highly conserved nature of this protein makes it necessary to use of long sequences for these phylogenetic comparisons and makes it more difficult to identify the temporal distribution of isolates during a single epizootic, within a restricted geographic region. The 5′ region of Seg-3 encodes part of the carapace domain (7–297aa) and can be used as an alternative to the full sequence for the prediction of the geographic origin of the various strains of BTV with good proximity. This region of Seg-3 can be determined directly by RT-PCR amplification and sequencing, potentially facilitating future epidemiological studies of BTV. Up to now all BTV incursions into south-eastern Europe, by viruses belonging to both the eastern (BTV-1, -9, -16) and western topotypes (BTV-4 eastern Mediterranean strain) were from a geographic area located to the east/southeast of Greece. Epidemiological and phylogenetic analysis data indicate that the Middle East potentially represents an important ecologic niche for these viruses, and a continuous source for BTV further incursions into Europe. The continuous circulation of different serotypes, including live vaccine strains, in this geographic region provides the conditions of genetic reassortment, and the generation of novel strains, potentially with altered biological properties. The detection of essentially the same Seg-3 in isolates belonging to different serotypes (i.e with divergent Seg-2 sequences), indicates that genome segment reassortment has occurred during the evolution of these strains, as analysed by Nomikou et al (manuscript in preparation).

## Supporting Information

Figure S1 Phylogenetic tree inferred with maximum likelihood analysis using the 5′ region of BTV Seg-3 (nucleotides 36 to 908) and the GTR + I + Γ model. The viruses included are indicated in tables 1 and 2. Isolate abbreviations: Serotype/origin (GRE: Greece, ISR: Israel, ZIM: Zimbabwe, NIG: Nigeria, SUD: Sudan, MOR: Morocco, SPA: Spain, TUN: Tunisia, ITL: Italy, CORS: Corsica, USA: USA, CYP: Cyprus, BUL: Bulgaria, TUR: Turkey, IND: India, AUSAsia: Australasia, INDO: Indonesia, TAIW: Taiwan, MAY: Malaysia, AUS: Australia, ISA: Indonesia, RSA: Reference strain)/year of isolation or IAH dsRNA virus reference collection number. The numbers indicated on branches are non-parametric bootstrap (NPB) probabilities and only values with P>0.6 are shown.
(2.50 MB TIF)

Figure S2 Phylogenetic tree inferred with maximum likelihood analysis using the middle region of BTV Seg-3 (nucleotides 909 to 1778) and the GTR + I + Γ model. The viruses included are indicated in tables 1 and 2. Isolate abbreviations: Serotype/origin (GRE: Greece, ISR: Israel, ZIM: Zimbabwe, NIG: Nigeria, SUD: Sudan, MOR: Morocco, SPA: Spain, TUN: Tunisia, ITL: Italy, CORS: Corsica, USA: USA, CYP: Cyprus, BUL: Bulgaria, TUR: Turkey, IND: India, AUSAsia: Australasia, INDO: Indonesia, TAIW: Taiwan, MAY: Malaysia, AUS: Australia, ISA: Indonesia, RSA: Reference strain)/year of isolation or IAH dsRNA virus reference collection number. The numbers indicated on branches are non-parametric bootstrap (NPB) probabilities and only values with P>0.6 are shown.
(2.49 MB TIF)

Figure S3 Phylogenetic tree inferred with maximum likelihood analysis using the 3′ region of BTV Seg-3 (nucleotides 1779 to 2648) and the GTR + I + Γ model. The viruses included are indicated in tables 1 and 2. Isolate abbreviations: Serotype/origin (GRE: Greece, ISR: Israel, ZIM: Zimbabwe, NIG: Nigeria, SUD: Sudan, MOR: Morocco, SPA: Spain, TUN: Tunisia, ITL: Italy, CORS: Corsica, USA: USA, CYP: Cyprus, BUL: Bulgaria, TUR: Turkey, IND: India, AUSAsia: Australasia, INDO: Indonesia, TAIW: Taiwan, MAY: Malaysia, AUS: Australia, ISA: Indonesia, RSA: Reference strain) /year of isolation or IAH dsRNA virus reference collection number. The numbers indicated on branches are non-parametric bootstrap (NPB) probabilities and only values with P>0.6 are shown.
(2.63 MB TIF)
