# Supplementary figures and images for: Evolution and Phylogenetic Analysis of Full-Length VP3 Genes of Eastern Mediterranean Bluetongue Virus Isolates
Source: PLoS One. 2009 Jul 30;4(7):e6437. doi: 10.1371/journal.pone.0006437 (PMC2713410; doi:10.1371/journal.pone.0006437)

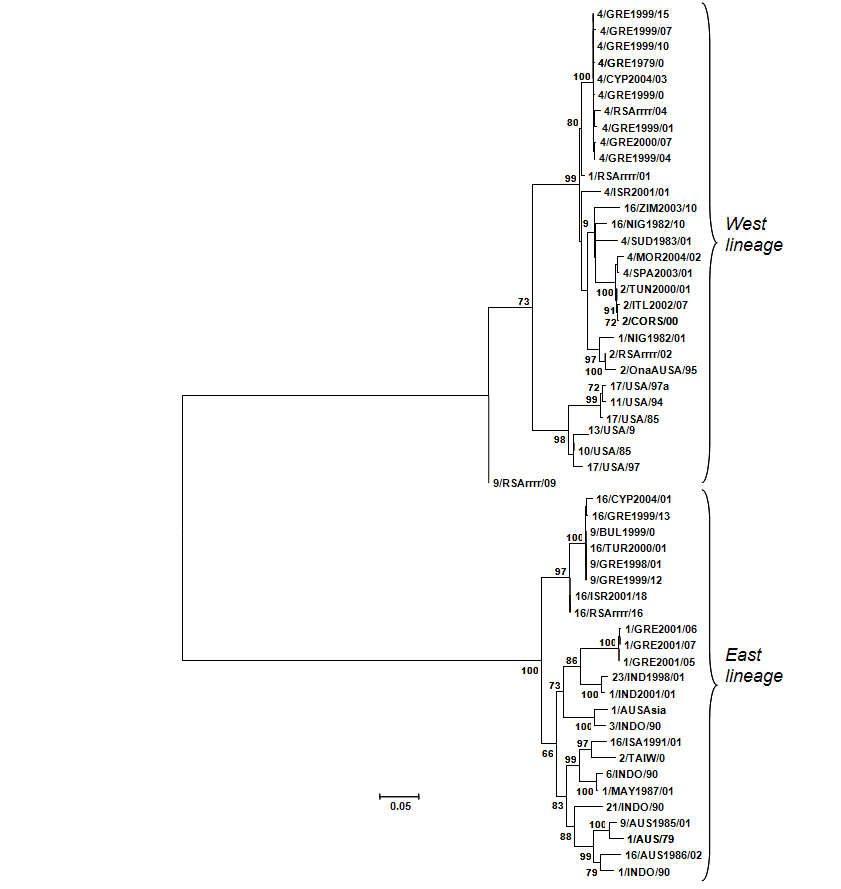

Supplement: Figure S1 — Phylogenetic tree inferred with maximum likelihood analysis using the 5′ region of BTV Seg-3 (nucleotides 36 to 908) and the GTR + I + Γ model. The viruses included are indicated in tables 1 and 2. Isolate abbreviations: Serotype/origin (GRE: Greece, ISR: Israel, ZIM: Zimbabwe, NIG: Nigeria, SUD: Sudan, MOR: Morocco, SPA: Spain, TUN: Tunisia, ITL: Italy, CORS: Corsica, USA: USA, CYP: Cyprus, BUL: Bulgaria, TUR: Turkey, IND: India, AUSAsia: Australasia, INDO: Indonesia, TAIW: Taiwan, MAY: Malaysia, AUS: Australia, ISA: Indonesia, RSA: Reference strain)/year of isolation or IAH dsRNA virus reference collection number. The numbers indicated on branches are non-parametric bootstrap (NPB) probabilities and only values with P>0.6 are shown. (2.50 MB TIF) [file pone.0006437.s001.tif]

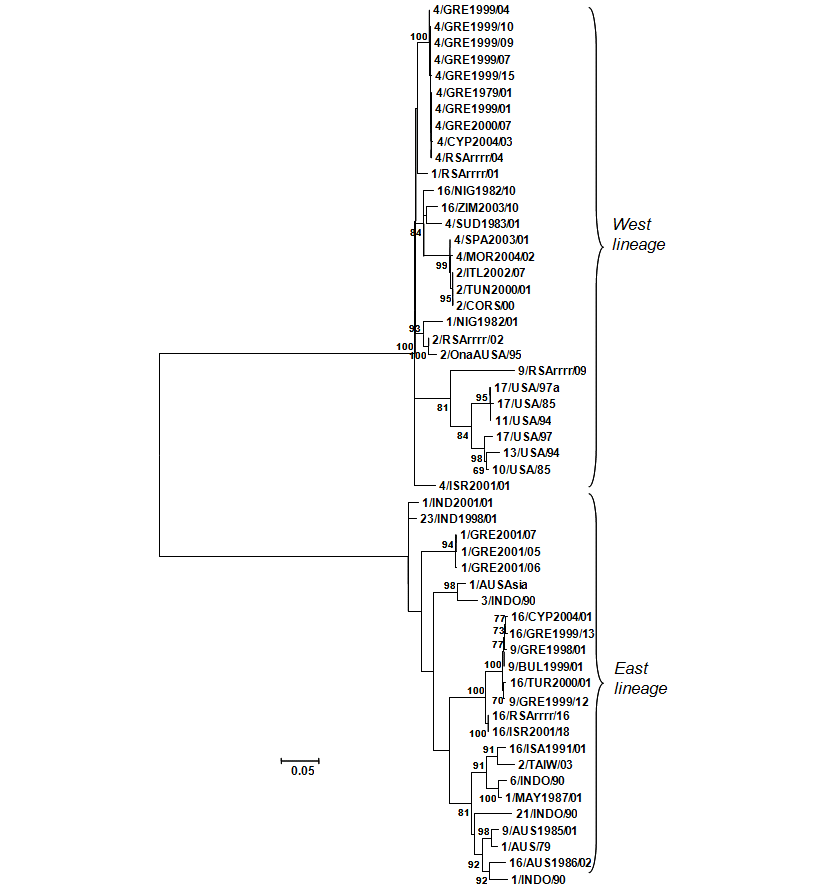

Supplement: Figure S2 — Phylogenetic tree inferred with maximum likelihood analysis using the middle region of BTV Seg-3 (nucleotides 909 to 1778) and the GTR + I + Γ model. The viruses included are indicated in tables 1 and 2. Isolate abbreviations: Serotype/origin (GRE: Greece, ISR: Israel, ZIM: Zimbabwe, NIG: Nigeria, SUD: Sudan, MOR: Morocco, SPA: Spain, TUN: Tunisia, ITL: Italy, CORS: Corsica, USA: USA, CYP: Cyprus, BUL: Bulgaria, TUR: Turkey, IND: India, AUSAsia: Australasia, INDO: Indonesia, TAIW: Taiwan, MAY: Malaysia, AUS: Australia, ISA: Indonesia, RSA: Reference strain)/year of isolation or IAH dsRNA virus reference collection number. The numbers indicated on branches are non-parametric bootstrap (NPB) probabilities and only values with P>0.6 are shown. (2.49 MB TIF) [file pone.0006437.s002.tif]

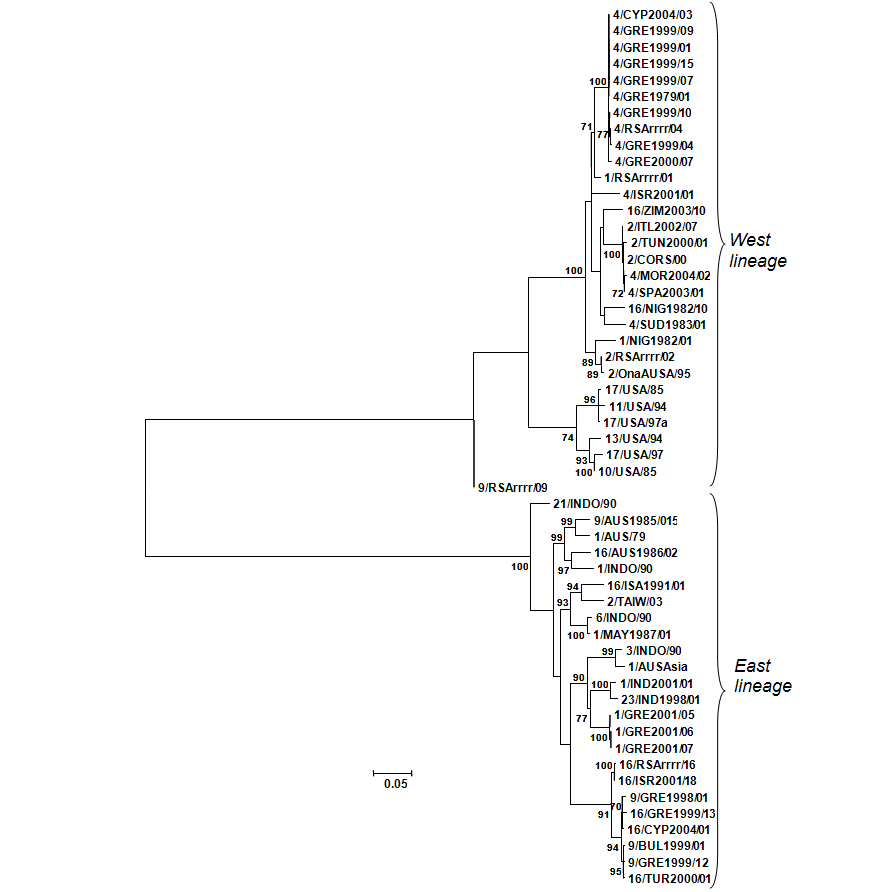

Supplement: Figure S3 — Phylogenetic tree inferred with maximum likelihood analysis using the 3′ region of BTV Seg-3 (nucleotides 1779 to 2648) and the GTR + I + Γ model. The viruses included are indicated in tables 1 and 2. Isolate abbreviations: Serotype/origin (GRE: Greece, ISR: Israel, ZIM: Zimbabwe, NIG: Nigeria, SUD: Sudan, MOR: Morocco, SPA: Spain, TUN: Tunisia, ITL: Italy, CORS: Corsica, USA: USA, CYP: Cyprus, BUL: Bulgaria, TUR: Turkey, IND: India, AUSAsia: Australasia, INDO: Indonesia, TAIW: Taiwan, MAY: Malaysia, AUS: Australia, ISA: Indonesia, RSA: Reference strain) /year of isolation or IAH dsRNA virus reference collection number. The numbers indicated on branches are non-parametric bootstrap (NPB) probabilities and only values with P>0.6 are shown. (2.63 MB TIF) [file pone.0006437.s003.tif]
